# A rare cause of sudden chest pain and dyspnea: A CARE-compliant case report of Chilaiditi syndrome

**DOI:** 10.1097/MD.0000000000020220

**Published:** 2020-05-15

**Authors:** Yanling Xu, Qi Wang, Guangping Meng, Dapeng Li, Zhiming Ma, Peng Gao, Jie Zhang, Qun Zhang, Zhenzhong Su

**Affiliations:** a Department of Respiratory Medicine; b Department of Geriatrics and General Medicine; c Departments of Respiratory Medicine, Affiliated Hospital of Jilin Medical University, Jilin, Jilin 132000; d Department of Gastrointestinal Nutrition and Hernia Surgery; e Department of Critical Care Medicine, The Second Affiliated Hospital of Jilin University, Changchun, Jilin 130041, China.

**Keywords:** chest pain, Chilaiditi syndrome, dyspnea

## Abstract

**Rationale:**

Chilaiditi syndrome is a rare disorder characterized by a broad spectrum of (gastro-intestinal) symptoms caused by interposition of a segment of bowel between the liver and the diaphragm. Most cases present with abdominal symptoms and the morbidity tend to increase with age.

**Patient concerns:**

Here we present a rare case of Chilaiditi syndrome. An elderly postmenopausal woman developed unresolved postoperative respiratory symptoms and chest pain. Chest auscultation revealed considerable attenuation of respiratory sounds. She showed postoperative increase in D-dimer level and sudden onset of dyspnea.

**Diagnoses:**

Considering the presence of atelectasis in the middle and lower lobes of the right lung, bedside fiberoptic bronchoscopy was performed immediately to rule out bronchial phlegm embolism. However, no phlegm embolism was found in the left lung, and a small amount of yellow–white mucus was seen in the upper lobe of the right lung. Due to external pressure, the lumen of the middle and lower lobes of the right lung was obviously narrowed.

**Interventions:**

The patient was placed in a semi-sitting position and a tube was passed through the anus to decompress the intestinal cavity; in addition, she received potassium supplementation.

**Outcomes:**

The patient's symptoms improved markedly. Chest and semi-supine abdominal plain radiographs showed enhanced lung markings, shadows in the left lower lung lobes, elevation of the right diaphragm, and small amount of pneumoperitoneum. The patient recovered after 5 days of continuous treatment and was discharged.

**Lessons:**

Emergency computed tomographic pulmonary angiography may facilitate the diagnosis of Chilaiditi syndrome, especially in the postoperative setting. Occurrence of Chilaiditi syndrome in this patient was likely associated with surgical factors. Appropriate investigations and clear identification of etiology are essential for successful treatment.

## 1 Introduction

Chilaiditi syndrome is characterized by hepato-diaphragmatic interposition of the bowel, which typically involves the transverse colon. The estimated global morbidity rate is 0.025% to 0.28%.^[1]^ Chilaiditi syndrome was first reported by the Greek scholar Demetrius Chilaiditi in 1910.^[2]^ It refers to the interposition of the colon through the anterior hepatic space or the posterior hepatic space between the liver and the diaphragm. The condition may be caused by congenital or acquired liver descent and abnormal location.^[3]^ For example, abnormal development of hepatic ligaments and/or hepatic lobes or cirrhotic atrophy of hepatic lobes may enlarge the hepatic diaphragm space and cause displacement of the transverse colon into the hepatic diaphragm space. Because of the relative lack of awareness of this disease, it is liable to be misdiagnosed as pulmonary embolism. Here we report a case of interpositional colon syndrome that presented as postoperative acute chest pain and dyspnea. Appropriate^[3]^ investigations facilitated prompt diagnosis and treatment.

## 2 Case report

**Figure 1:**
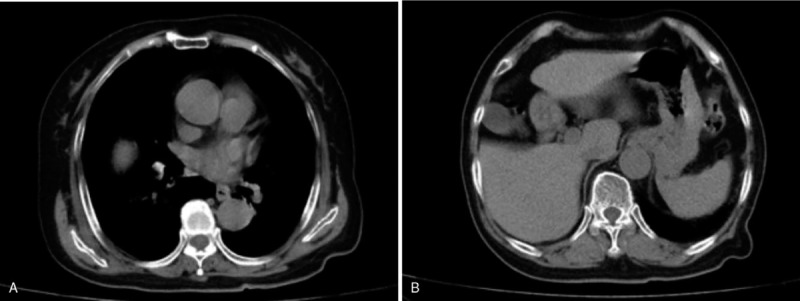
Preoperative CT shows no abnormality of diaphragm or colon (A and B).

**Figure 2:**
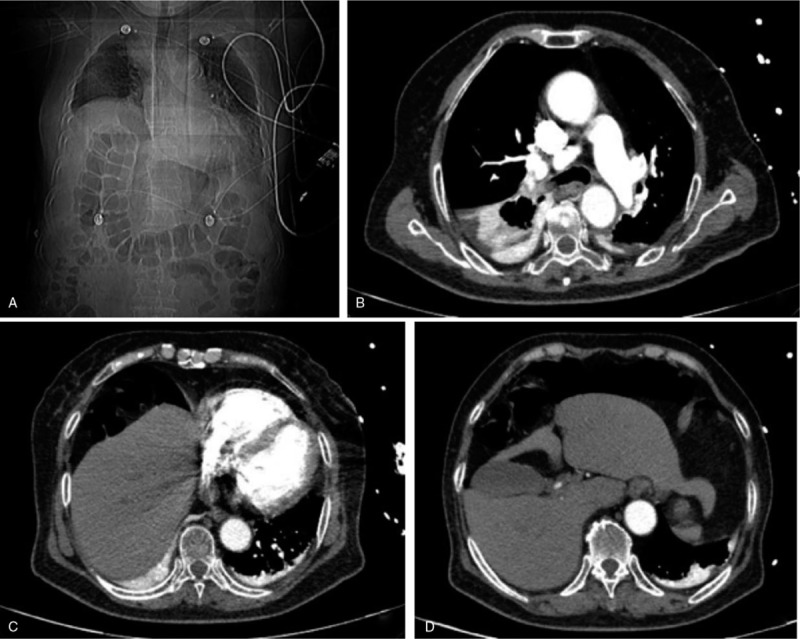
CT showing no signs of pulmonary embolism, leg vein thrombosis, or patchy lung consolidation (A and B). There is upward displacement of the diaphragm along with left-sided shift of the mediastinum (C). The colon is seen located between the liver and the diaphragm (D).

A 66-year-old woman with cervical squamous cell carcinoma was admitted to the department of gynecology on October 16, 2018. Preoperative chest computed tomography showed no abnormality of diaphragm or colon (Fig. 1A and B). On the second day after the operation, the patient had no abdominal distension and was asked to get out of bed as a preventive measure against thrombosis. At 19:00 hours on the same day, the patient developed acute right chest pain and dyspnea; there was no nausea or vomiting. Her percutaneous oxygen saturation showed a progressive decrease with a minimum level of 75%. Chest auscultation revealed considerable attenuation of respiratory sounds. The results of arterial blood gas analysis were: PO_2_ 43 mm Hg; PCO_2_ 39 mm Hg; D-dimer 1.13 μg/mL; K^+^ 3.19 mmol/L; Ca^2+^ 1.97 mmol/L; P 0.58 mmol/L; Mg^2+^ 0.70 mmol/L. Postoperative increase in D-dimer level and sudden development of dyspnea in an elderly postmenopausal woman with tumor was highly suspicious of pulmonary embolism; therefore, emergency computed tomographic pulmonary angiography (CTPA) was performed. However, no signs of pulmonary embolism, thrombosis in the leg veins, or patchy lung consolidation were observed (Fig. 2A and B). At the same time, there was upward displacement of the diaphragm along with left-sided shift of the mediastinum (Fig. 2C); in addition, the colon was found located between the liver and the diaphragm (Fig. 2D). Considering the presence of atelectasis in the middle and lower lobes of the right lung, bedside fiberoptic bronchoscopy was performed immediately to rule out bronchial phlegm embolism. However, no phlegm embolism was found in the left lung, and a small amount of yellow–white mucus was seen in the upper lobe of the right lung. Due to external pressure, the lumen of the middle and lower lobes of the right lung was obviously narrowed. Based on the patient's symptoms and the results of CTPA, the diagnosis of Chilaiditi syndrome was established. Written informed consent of the patient was obtained for publication of this case report. This case study was approved by the ethics committee of The Second Affiliated Hospital of Jilin University.

**Figure 3:**
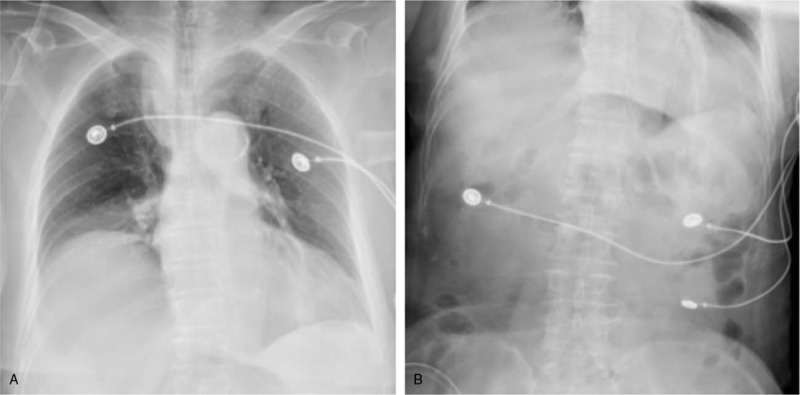
Chest (A) and semi-supine abdominal plain (B) radiographs showing enhanced lung markings, shadows in the left lower lung lobes, elevation of the right diaphragm, and mild pneumoperitoneum.

The patient was placed in a semi-sitting position and a tube was passed through the anus to decompress the intestinal cavity; in addition, she received potassium supplementation. The patient's symptoms improved markedly from October 21, 2018. Bedside chest and semi-supine abdominal plain radiographs (Fig. 3A and B) showed enhanced lung markings, shadows in the left lower lung lobes, elevation of the right diaphragm, and small amount of pneumoperitoneum. Her serum K^+^ level was 4.0 mmol/L. The patient recovered after 5 days of continuous treatment and was discharged.

We also performed a literature search for similar case reports using the term Chilaiditi syndrome, and identified 13 other cases of Chilaiditi syndrome (mean age of patients: 56.4 years, 8 males, and 5 females). These cases were reported from America, Egypt, China, India, Turkey, Italy, Singapore, and Japan.^[4,5,6,7,8,9,10,11,12,13]^ The clinical features ranged from lack of symptoms to abdominal pain, flatulence, decreased appetite, nausea, vomiting, constipation, dyspnoea, and chest pain (Table 1).

**Table 1:**
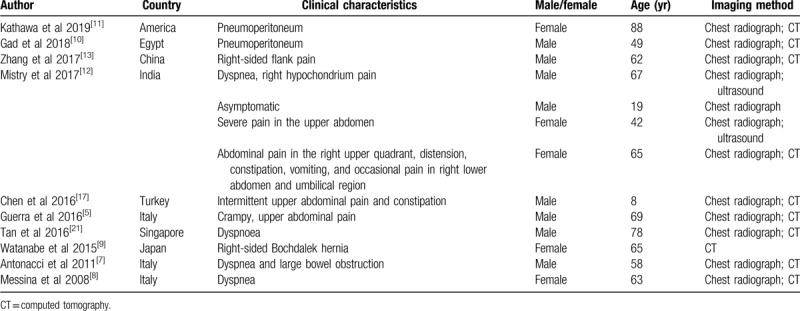
Summary of published case reports of Chilaiditi syndrome.

## 3 Discussion

Chilaiditi syndrome or subphrenic interposition of the colon is a rare condition with a predilection for male (male to female ratio, 4:1).^[1]^ Chilaiditi syndrome is incidentally detected in 0.025% to 0.28% of chest and abdominal plain films^[14,15]^ and 1.18% to 2.4% of abdominal computed tomography scans.^[15]^ The main radiological sign is appearance of intra-abdominal free air. In this case, CT examination of the chest revealed the presence of this disease. Conditions that lead to enlarged right subdiaphragmatic space or hypermobility of the intestines may cause Chilaiditi syndrome. Various factors may predispose to Chilaiditi syndrome including diaphragmatic, intestinal, hepatic, and other factors. In addition, iatrogenic factors may also lead to Chilaiditi syndrome.^[14,16]^ A previous study suggested that abdominal pain with subphrenic free air may not always suggest surgical emergency. A combination of physical examination and imaging assessment may help arrive at a correct diagnosis and avoid unnecessary surgery.^[17]^ In the case report by Gad et al, Chilaiditi syndrome was diagnosed by computed tomography; in addition, the authors highlighted the importance of physical examination in assessing patients with apparent air under the diaphragm.^[10]^

In our patient, the condition was related to gynecological operation. The clinical symptoms of Chilaiditi syndrome are associated with respiratory and digestive systems such as abdominal pain, constipation, vomiting, respiratory distress, anorexia, volvulus, or intestinal obstruction. One patient developed intestinal perforation. Conservative management is often sufficient in children with symptomatic Chilaiditi syndrome.^[18]^ The precise etiopathogenetic mechanism of Chilaiditi syndrome is not clear,^[19]^ and it is mainly associated with congenital and acquired factors. The diagnosis of Chilaiditi syndrome seems to be relatively straightforward because the interposition of the colon (the so-called Chilaiditi sign) is observable on chest films, abdominal plain films, or B-ultrasound; however, this sign can be present temporarily or permanently.^[20]^ Previous case reports suggest that Chilaiditi syndrome may appear at any age; dyspnoea and upper abdominal pain are commonly reported initial symptoms. Chest radiograph and CT are helpful in the diagnosis.^[4,5,6,7,8,9,10,11,12,13]^

The treatment strategy for Chilaiditi syndrome mainly includes conservative management with nasogastric decompression, administration of stool softeners and enemas, and intravenous fluids. Conservative treatment is the first choice for most patients, unless the condition occurs in a surgical setting. Our patient was also conservatively treated. This treatment has been shown to be effective.

Our patient did not exhibit a chronic disease course; she developed postoperative acute dyspnea and right chest pain. Pulmonary embolism (PTE) was highly suspected based on the history of surgery, dyspnea, chest pain, and elevated D-dimer level. However, for patients with unresolved respiratory symptoms and chest pain, emergency CTPA can facilitate accurate diagnosis, especially in patients after operation.

## 4 Conclusion

In conclusion, we present a rare case of Chilaiditi syndrome and highlight the key aspects of diagnosis and treatment. Maintenance of electrolyte balance, physical activity, and consumption of high-fiber diet help promote intestinal peristalsis and resolve the symptoms. Emergency CTPA may facilitate the diagnosis of Chilaiditi syndrome, especially in the postoperative setting. In addition, we believe that the occurrence of Chilaiditi syndrome in this patient was associated with surgical factors. Finally, appropriate investigations and clear etiology are essential for successful treatment.

## Author contributions

**Conceptualization:** Yanling Xu, Zhenzhong Su.

**Data curation:** Yanling Xu, Qi Wang, Guangping Meng, Dapeng Li, Zhiming Ma, Peng Gao, Jie Zhang, Qun Zhang, Zhenzhong Su.

**Funding acquisition:** Yanling Xu, Zhenzhong Su.

**Investigation:** Yanling Xu, Qi Wang, Dapeng Li, Zhiming Ma, Peng Gao, Jie Zhang, Qun Zhang, Zhenzhong Su.

**Methodology:** Zhenzhong Su.

**Project administration:** Yanling Xu.

**Resources:** Qi Wang, Guangping Meng, Dapeng Li, Zhiming Ma, Peng Gao, Jie Zhang, Qun Zhang.

**Supervision:** Yanling Xu, Zhenzhong Su.

**Validation:** Yanling Xu, Qi Wang, Guangping Meng, Dapeng Li, Zhiming Ma, Peng Gao, Jie Zhang, Qun Zhang, Zhenzhong Su.

**Visualization:** Yanling Xu, Qi Wang, Guangping Meng, Dapeng Li, Zhiming Ma, Peng Gao, Jie Zhang, Qun Zhang, Zhenzhong Su.

**Writing – original draft:** Yanling Xu, Qi Wang, Guangping Meng, Dapeng Li.

**Writing – review & editing:** Yanling Xu, Qi Wang, Guangping Meng, Dapeng Li, Zhiming Ma, Peng Gao, Jie Zhang, Qun Zhang, Zhenzhong Su.
